# Distinct immune responses to HIV and CMV in Hofbauer cells across gestation highlight evolving placental immune dynamics

**DOI:** 10.3389/fimmu.2026.1832988

**Published:** 2026-06-09

**Authors:** Viviane Schuch, Daniel Hossack, Tiffany Hailstorks, Rana Chakraborty, Erica L. Johnson

**Affiliations:** 1 Department of Microbiology, Biochemistry, and Immunology, Morehouse School of Medicine, Atlanta, GA, United States; 2 Department of Gynecology and Obstetrics, Emory University School of Medicine, Atlanta, GA, United States; 3 Department of Pediatrics, Division of Pediatric Infectious Disease, University of Miami, Miller School of Medicine, Miami, FL, United States; 4 Department of Obstetrics and Gynecology, Morehouse School of Medicine, Atlanta, GA, United States

**Keywords:** cytomegalovirus, gestational immunity, HIV, Hofbauer cells, interferon, maternal–fetal interface, placenta

## Abstract

**Background:**

Hofbauer cells are fetal macrophages in the placental villous core that contribute to maternal–fetal tolerance and antiviral defense, but how their immune programs change across gestation in response to viral exposure remains poorly understood.

**Methods:**

Primary Hofbauer cells were isolated from early- to mid-gestation and term human placentas and exposed *in vitro* for 24 h to HIV-1 or cytomegalovirus (CMV), together with mock controls. Transcriptional responses were profiled by bulk RNA sequencing using differential expression, pathway enrichment, and co-expression network analyses, integrated with placenta-enriched gene annotations from the Human Protein Atlas. Secreted cytokines were quantified using a multiplex bead assay.

**Results:**

Gestational stage strongly shaped both the transcriptional landscape and the magnitude of antiviral responses. At term, both HIV-1 and CMV induced a shared interferon-rich antiviral program, but CMV uniquely coupled this response to broad repression of mitochondrial and lipid metabolism, extracellular matrix and junctional pathways, and multiple placenta-enriched structural and immune genes. CMV exposure at term was also associated with remodeling of WNT signaling characterized by altered expression of Frizzled receptors and induction of the negative regulator *RNF43*, consistent with receptor-level feedback control of the pathway. In contrast, HIV-1 at term preserved proliferative, antigen-presentation, and T-cell co-stimulation signatures. In early- to mid-gestation Hofbauer cells, viral exposure did not yield significant DEGs but showed coordinated pathway-level changes, with HIV-1 favoring interferon-associated signaling and CMV preferentially altering metabolic and remodeling pathways.

**Conclusion:**

Gestational stage establishes the immune–metabolic baseline of Hofbauer cells and strongly shapes their responses to viral exposure. At term, HIV and CMV converge on a shared interferon-driven antiviral program, but CMV uniquely couples this response to coordinated disruption of metabolic, structural, and signaling pathways in Hofbauer cells. These findings suggest that CMV drives a metabolically and structurally constrained antiviral state, providing a mechanistic framework linking CMV exposure to placental vulnerability and enhanced HIV susceptibility at the maternal–fetal interface.

## 1 Introduction

The placenta is a dynamic and multifunctional organ that supports fetal development and maternal adaptation during pregnancy. As the primary interface between mother and fetus, the placenta facilitates the exchange of nutrients and gases, the elimination of waste products, and the production of hormones critical to maintaining gestation and fetal growth (1). Additionally, the placenta acts as a selective barrier, providing immunological protection to the fetus against invasive pathogens while supporting maternal immune tolerance (2). Despite these defense mechanisms, exposure to viral pathogens such as HIV and CMV can significantly perturb placental development and immune homeostasis, even in the absence of direct vertical transmission, leading to adverse pregnancy outcomes, including preeclampsia, fetal growth restriction, and preterm birth (3–5). These disruptions have long-term implications for offspring health, yet the placenta remains one of the most understudied organs, particularly regarding how its immune landscape evolves across gestation and the impact of viral exposure during pregnancy (6, 7). Understanding the mechanisms and timing of viral transmission is crucial for improving maternal-fetal health outcomes and may help develop targeted therapies.

Hofbauer cells (HCs), the fetal-derived macrophages of the placenta, play a critical role in mediating immune tolerance and defense at the maternal-fetal interface. These cells are present as early as 18 days post-conception and persist throughout gestation (8–10). HCs comprise a heterogeneous population of M2a, M2b, and M2c phenotypes (11, 12), characterized by surface marker expression, including CD163, CD206, CD209, CD4, *CCR5*, and *CXCR4* (8, 13). These placental macrophages maintain an anti-inflammatory milieu through secretion of IL-10 and TGF-β (13) and retain plasticity that enables adaptation to environmental cues (8). Our group and others have shown that early- and mid-gestation HCs exhibit robust antiviral and inflammatory responses to stimuli such as interferon (IFN)-γ and RIG-I agonists, whereas term HCs display a dampened antiviral profile and enhanced M2 polarization (8, 14). This temporal shift in phenotype suggests that placental immunity is developmentally regulated and may contribute to the timing of viral transmission during gestation.

Vertical transmission of HIV and CMV provides a powerful model to understand the age-dependent regulation of placental immunity. Clinical and epidemiological data demonstrate that the risk of *in utero* viral transmission rises dramatically with gestational age, with 40-70% occurring during the third trimester (15–17). On the other hand, infections acquired earlier in pregnancy, though less frequently transmitted, are associated with more severe fetal outcomes (18, 19). At the maternal-fetal interface, HCs are key targets for HIV and CMV exposure (13, 20) and act as potential reservoirs for viral persistence and transfer across the placental barrier (13, 21–23). Our prior studies demonstrate that early/mid-gestation HCs mount robust antiviral responses to HIV characterized by activation of RIG-I and MDA-5 pathways, type I and III interferon production, and induction of interferon-stimulated genes (ISGs) (14). These antiviral states may restrict viral replication and protect the developing fetus. In contrast, term HCs exposed to HIV or CMV display attenuated type I/III IFN signaling, reduced *STAT1/STAT2* activation, and elevated *STAT5* phosphorylation, which correlated with enhanced HIV replication (14, 24). This functional shift likely reflects developmental immune reprogramming within the placenta and may explain why vertical transmission peaks at the end of gestation. Thus, HIV and CMV may exploit gestational immunological windows of reduced antiviral capacity to facilitate transmission.

Despite significant advances in understanding placental immune regulation, little is known about how HCs’ immune responses evolve across gestation, particularly in the context of viral infections. In this study, we investigated how HIV and CMV exposure remodel the transcriptional landscape of HCs across gestation. We examined differentially expressed genes (DEGs), signaling pathways, co-expression modules, and placenta-enriched gene annotations in early/mid-gestation versus term HCs following viral exposure, alongside mock-exposed controls, and integrated these data with cytokine profiles. Our goal was to define how gestational age and viral identity jointly modulate immune balance, placental developmental programs, and vulnerability to pregnancy complications.

## 2 Results

### 2.1 Overview of experimental design and datasets

**Figure 1:**
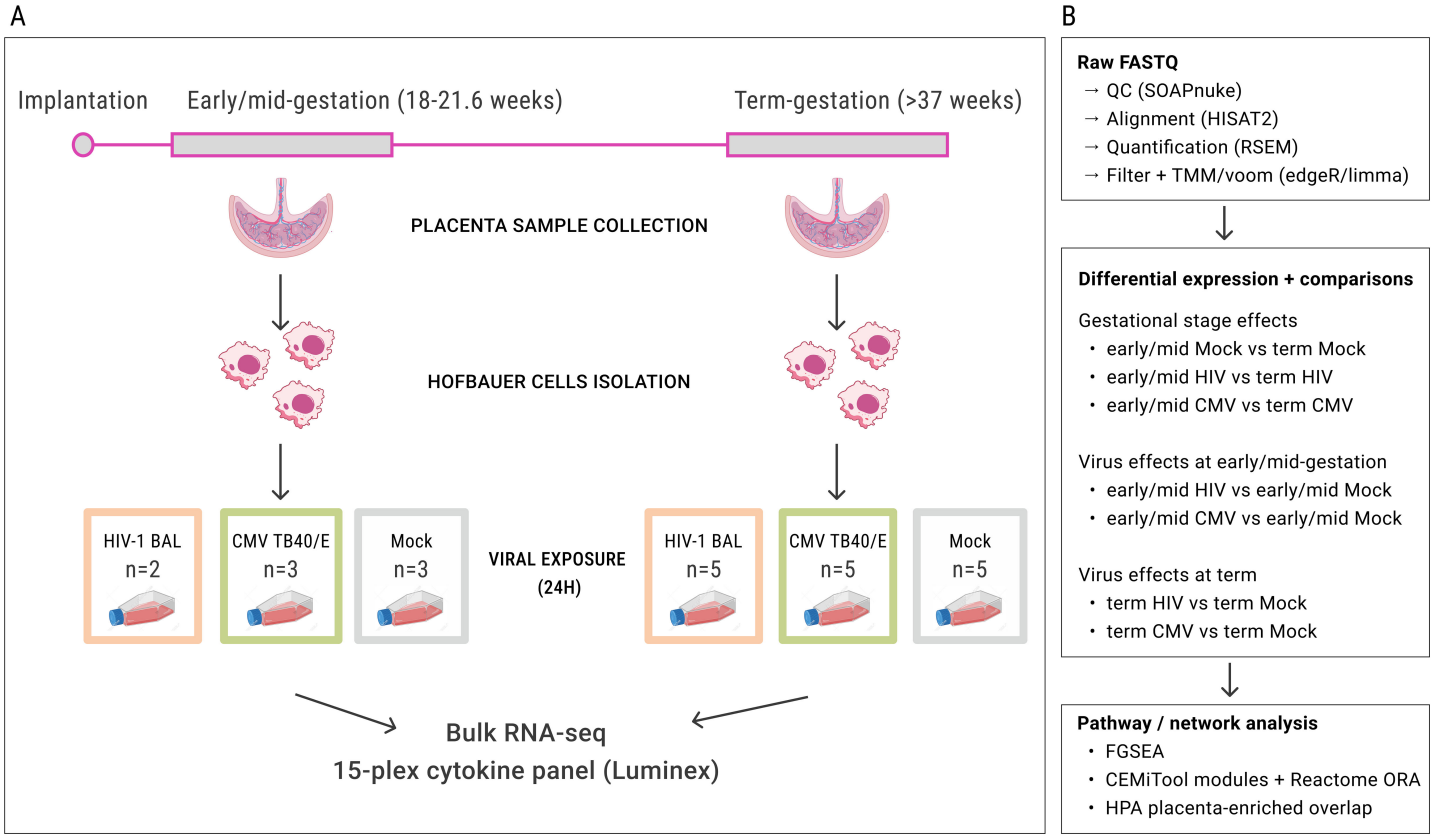
Experimental design and analysis workflow. **(A)** Schematic of sample collection and *in vitro* experiments. **(B)** Overview of the RNA-seq analysis.

HCs were isolated from human placentas at early/mid-gestation (18–21.6 weeks) and term (>37 weeks) and exposed for 24 hours to HIV-1 BaL or CMV TB40/E, alongside gestational age–matched mock-exposed controls (**Figure 1A**; **Supplementary Table 1**). Transcriptional responses were quantified by bulk RNA sequencing, and cytokine and chemokine secretion were measured in culture supernatants using a 15-plex Luminex panel.

RNA-seq data were analyzed to assess global transcriptional structure, differential gene expression, pathway activity, and co-expression patterns across gestational stage and viral exposure (**Figure 1B**). Differentially expressed genes were further annotated for placenta-enriched expression to identify virus- and stage-specific perturbations relevant to placental biology. Cytokine profiles were analyzed across all six experimental conditions and interpreted in parallel with transcriptomic signatures.

### 2.2 Gestational stage and viral exposure shape global transcriptional structure

**Figure 2:**
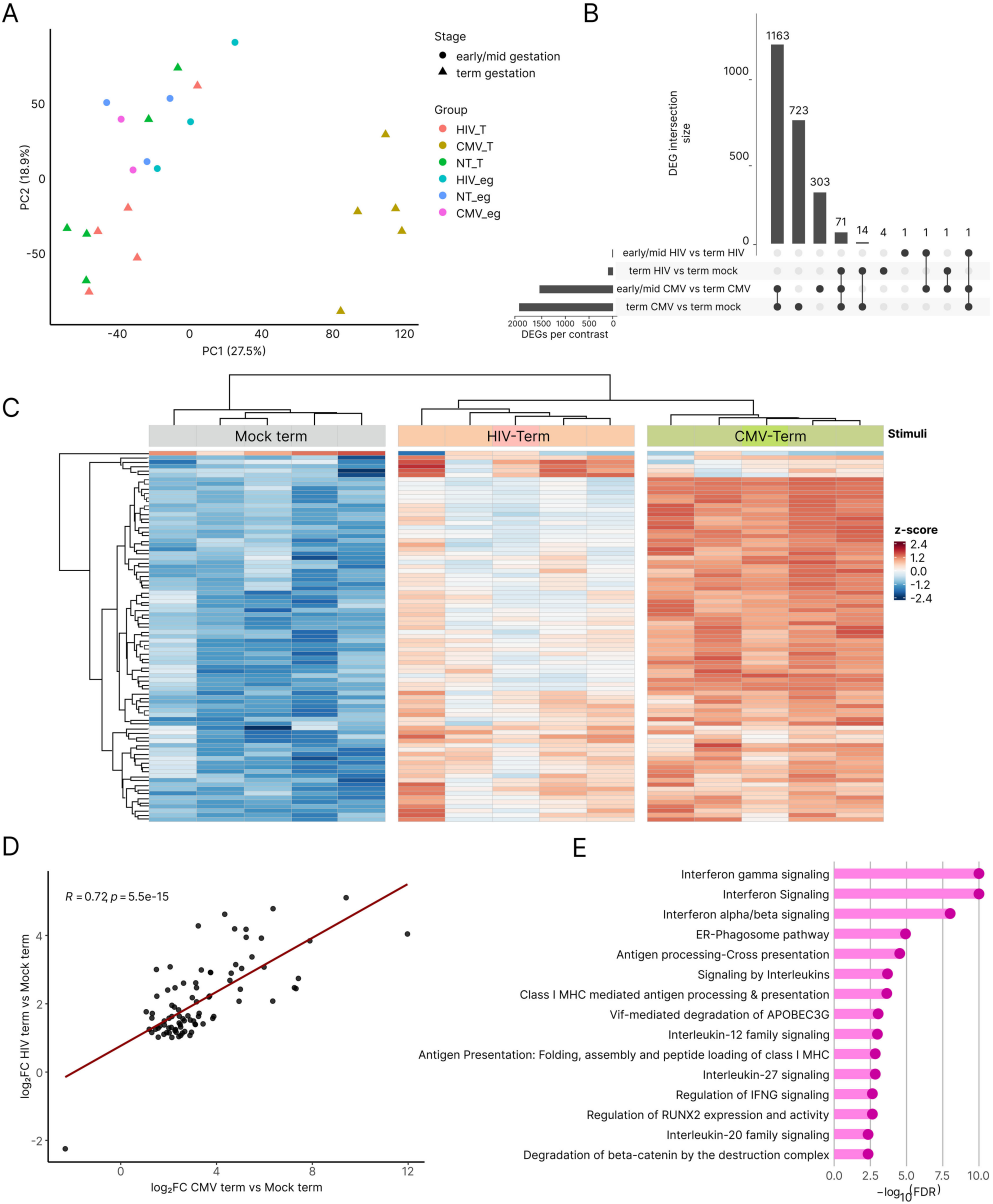
Global transcriptional structure, DEG landscape, and shared signature between term CMV and term HIV. **(A)** Principal component analysis of batch-corrected transcriptomes, colored by virus (HIV, CMV, mock-exposed) and shaped by gestational stage (early/mid-gestation vs term-gestation). **(B)** UpSet plot showing overlaps between DEGs between contrasts with significant results (early/mid vs term within each virus and virus vs mock-exposed at term; FDR < 0.05, |log_2_FC| > 1). Vertical bars indicate intersection sizes; horizontal bars show the total number of DEGs per contrast. **(C)** Heatmap of z-score–scaled expression for significantly DEGs in both term CMV vs term mock-exposed and term HIV vs term mock-exposed. Columns are individual term samples, annotated by stimulus (term HIV, term CMV, term mock-exposed); rows are genes. **(D)** Concordance of log_2_ fold-change estimates for the shared DEGs in term-gestation CMV vs term mock-exposed (x-axis) and term HIV vs term mock-exposed (y-axis). Each point represents one gene; the line shows the linear regression fit, with Pearson correlation coefficient **(R)** and P-value indicated. **(E)** Over-representation analysis of shared up-regulated DEGs, showing significantly enriched pathways (Reactome) ranked by –log_10_(FDR).

PCA of batch-corrected, voom-normalized expression values from the 5,000 most variable genes defined the global organization of the HC transcriptome across gestational and viral conditions. PC1 accounted for 27.5% of the variance and separated term CMV-exposed HCs from all other groups, indicating that CMV exposure at term produces the most substantial transcriptional perturbation (**Figure 2A**). PC2, which explained 18.9% of the variance, captured additional stage- and treatment-associated structure among the remaining samples. PERMANOVA on Euclidean distances confirmed that both gestational stage and experimental group significantly contributed to transcriptome-wide variation (Stage: R² = 0.11, F = 3.29, p = 0.0018; Group: R² = 0.32, F = 2.37, p = 0.0001), and dispersion tests indicated that these differences were not attributable to within-group heterogeneity. Together, these analyses show that gestational stage establishes a baseline transcriptional framework that is subsequently remodeled by viral exposure, with CMV exposure at term exerting the strongest global effect.

### 2.3 Differential gene expression analysis

We fit limma–voom linear models to the normalized expression matrix and tested contrasts capturing virus effects within gestational stage, gestational-stage effects within exposure condition, and direct virus-versus-virus differences within gestational stage. Differential expression analysis revealed a strong dependence on both gestational stage and viral identity. At early/mid gestation, neither HIV nor CMV exposure induced genes meeting the DEG threshold (FDR < 0.05, |log_2_FC| > 1) when compared to gestationally matched mock controls, but pathway-level analyses revealed coordinated, sub-threshold transcriptional shifts, particularly in the HIV contrast. Similarly, comparison of early/mid and term mock-exposed HCs identified no significant DEGs.

In contrast, term HCs exhibited robust transcriptional responses to viral exposure, with CMV inducing a markedly larger DEG set than HIV. Specifically, term CMV exposure yielded 1,972 DEGs relative to term mock-exposed controls, whereas term HIV exposure resulted in 90 DEGs. Consistent with this pattern, comparison of early/mid and term CMV-exposed HCs identified 1,540 DEGs, indicating extensive gestational-stage remodeling of transcriptional programs in the context of CMV exposure. In contrast, the HIV gestational comparison identified only three DEGs, suggesting minimal stage-dependent transcriptional changes during HIV exposure. An UpSet analysis (**Figure 2B**) confirmed that the majority of DEGs were confined to CMV-related contrasts, particularly those involving term gestation, whereas HIV-associated DEG sets were comparatively small and showed limited overlap with CMV-driven transcriptional changes.

To directly test virus-specific transcriptional differences between the two viral exposures, we additionally performed within-stage CMV-versus-HIV contrasts. The direct term CMV versus term HIV comparison identified 1,615 DEGs at FDR < 0.05 and |log_2_FC| > 1, including 941 genes higher in term CMV-exposed HCs and 674 genes higher in term HIV-exposed HCs. In contrast, the analogous early/mid-gestation CMV versus HIV comparison identified no DEGs at the same threshold. Thus, direct virus-versus-virus testing further supported that CMV and HIV responses diverge most strongly at term, consistent with broader CMV-associated transcriptional remodeling in late-gestation HCs.

### 2.4 A shared interferon antiviral signature at term gestation is accompanied by virus-specific divergence

To test whether HIV and CMV converge on common antiviral programs at term, we intersected DEGs from the term CMV versus term mock comparison with those from the term HIV versus term mock comparison, yielding 85 shared genes. Of these, 84 were up-regulated, whereas *ARG2* was the only down-regulated gene. Hierarchical clustering of these 85 genes (row-wise z-scores) clearly separated term mock, term HIV, and term CMV into three distinct groups, with term CMV showing the strongest signal across the shared gene set (**Figure 2C**). Fold-change values for the shared genes were tightly correlated between term CMV and term HIV conditions (Pearson r = 0.72, p = 5.5 × 10_15_; **Figure 2D**), indicating that both viruses activate a broadly similar antiviral program at term, but with greater amplitude in CMV-exposed cells.

Functional annotation revealed that this antiviral program includes IFN–JAK–STAT signaling genes (*STAT1, STAT2, JAK2, IRF1, ETV7, DDX60, ZBP1, IFI35, SOCS1*), along with a large panel of ISG-like effector genes (*APOBEC3F, APOBEC3G, IFITM1, GBP1, GBP2, GBP3, GBP4, GBP5, TRIM22, APOL1, APOL2, APOL3, APOL6, SAMD9L, WARS1, PLAAT4*). Shared responses also include genes involved in antigen presentation and immunoproteasome (*TAP1, TAP2, PSMB8, PSMB9, PSMB10, PSME2, NLRC5, RFX5, HLA-F, HLA-E, CD74*), cytokine and chemokine signaling (*CXCL10, CXCL11, IL27, IL15RA, TNFSF10* (TRAIL), *TNFSF13B* (BAFF), *FLT3LG, SERPING1, FGL2*), T-cell and immune regulatory surface molecules (*BTN3A1, BTN3A3, BTN2A2, GIMAP4, GIMAP8, SECTM1, SEMA4D, VAMP5*), and metabolic and functional modulators (*PFKP, LAP3, LGALS3BP, PLA2G4A*, with *ARG2* downregulated).

Reactome overrepresentation analysis of the shared term-gestation signature revealed strong enrichment of IFN-α/β and -γ signaling, IL-27 and IL-12 cytokine pathways, MHC class I antigen processing/presentation modules (TAP-dependent peptide loading, immunoproteasome subunits, *NLRC5/RFX5* regulation), and viral restriction pathways including Vif-mediated degradation of *APOBEC3G* (**Figure 2E**; **Supplementary Table 4**). These results confirm that both viruses converge on a common IFN-driven antiviral program in HCs characterized by robust JAK–STAT activation, antiviral effector induction, and enhanced antigen presentation capacity, reinforcing their role as central coordinators of antiviral immunity at the maternal–fetal interface.

We next used the direct term CMV versus term HIV contrast to define pathway-level differences between the two viral exposures. Hallmark FGSEA showed that term CMV-exposed HCs were strongly enriched for interferon alpha response, interferon gamma response, TNFα signaling via NFκB, and inflammatory response pathways relative to term HIV-exposed HCs. In contrast, term HIV-exposed HCs were relatively enriched for oxidative phosphorylation, MYC targets, mTORC1 signaling, PI3K–AKT–mTOR signaling, adipogenesis, fatty acid metabolism, peroxisome, glycolysis, and epithelial–mesenchymal transition. Reactome analysis confirmed enrichment of interferon alpha/beta signaling, DDX58/IFIH1-mediated induction of interferon alpha/beta, TRAF6-mediated IRF7 activation, regulation of IFNA/IFNB signaling, and interferon gamma signaling in term CMV-exposed HCs. Conversely, pathways relatively enriched in term HIV-exposed HCs included translation, rRNA processing, amino acid–stress responses, cellular response to starvation, and related biosynthetic pathway annotations. Together, these results show that although CMV and HIV converge on a shared interferon-rich antiviral core at term, CMV elicits a more IFN-dominant inflammatory state, whereas HIV preserves comparatively stronger metabolic, translational, growth-associated, and remodeling-associated transcriptional programs.

Full differential expression and pathway enrichment results for the direct CMV-versus-HIV contrasts are provided in **Supplementary Tables 2**, **3**, and **7**, and the direct term CMV versus term HIV comparison is summarized in **Supplementary Figure 1**.

### 2.5 Virus- and stage-specific hallmark programs

**Figure 3:**
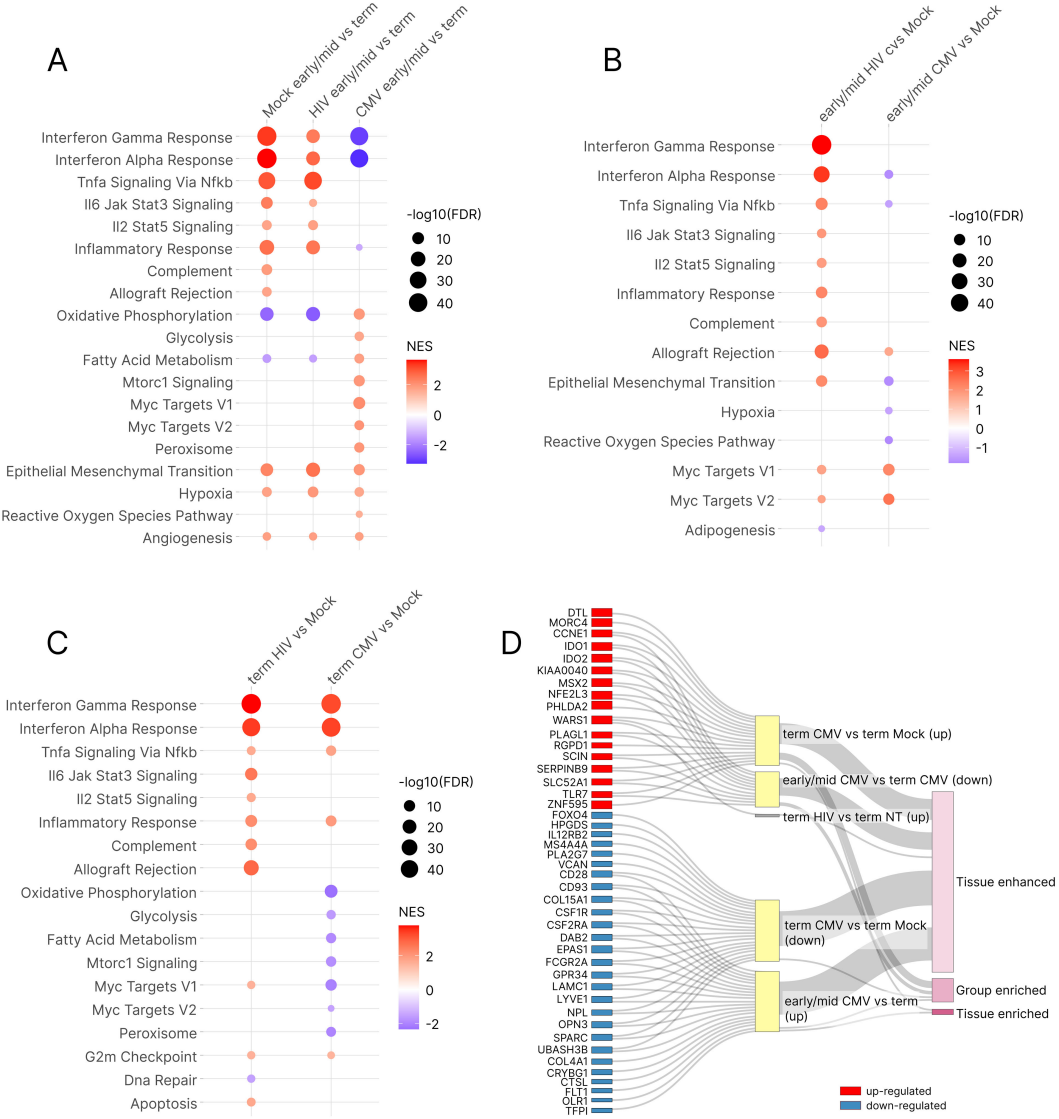
Gestational-stage and virus-specific modulation of placental macrophage pathways. **(A)** Gestational stage effect on Hallmark pathways. Dot plot summarizing pathway enrichment (MSigDB Hallmark gene sets) for early/mid–gestation versus term Hofbauer cells under three conditions (mock-exposed, HIV, CMV). Dot color represents the normalized enrichment score (NES), where red indicates pathway upregulation and blue indicates downregulation. Dot size reflects statistical significance (−log_10_(FDR)). **(B)** Virus-specific responses at early/mid gestation. Differential pathway activity for early/mid HIV vs early/mid mock-exposed and early/mid CMV vs early/mid mock-exposed. Early gestation Hofbauer cells exhibited strong interferon-driven responses to HIV, whereas CMV induced more modest changes. **(C)** Virus-specific responses at term gestation. Enrichment analysis for term HIV versus term mock-exposed and term CMV versus term mock-exposed. Both viruses triggered broad interferon-dominant responses at term, consistent with the shared antiviral core defined by overlapping DEGs. **(D)** Placenta-enriched proteins selectively perturbed by CMV at term gestation. Differentially expressed genes were intersected with Human Protein Atlas annotations for placenta-enhanced/placenta-enriched proteins, and their distribution across contrasts was visualized in a three-layer Sankey diagram. The left nodes show the 44 genes encoding placenta-enhanced or placenta-enriched proteins, the middle nodes represent the term CMV vs term mock-exposed, term HIV vs term mock-exposed, and early/mid CMV vs term CMV contrasts, and the right nodes indicate Human Protein Atlas tissue-enrichment classes (tissue-enhanced, group-enriched, tissue-enriched).

We performed Hallmark gene-set enrichment analysis (FGSEA) on ranked t-statistics for each contrast (**Figures 3A–C**). Across conditions, gestational stage emerged as the dominant determinant of pathway-level organization in HCs.

In both mock and HIV-exposed cells, early/mid-gestation HCs displayed a shared transcriptional profile characterized by enrichment of interferon and inflammatory programs, including IFN-α and IFN-γ responses, TNFα signaling via NFκB, IL6–JAK–STAT3, and IL2–STAT5 signaling (**Figure 3A**). In contrast, term mock and term HIV were enriched for metabolic pathways such as oxidative phosphorylation and fatty acid metabolism, consistent with a developmental transition from an interferon-high, metabolism-low state at early/mid-gestation to a more metabolically active state at term. Whereas HIV exposure did not substantially alter this gestational trajectory, CMV exposure revealed a distinct pattern. In comparisons of early/mid-gestation versus term CMV-exposed HCs, interferon pathways (IFN-α and IFN-γ responses) were downregulated at early/mid-gestation, while metabolic programs—including oxidative phosphorylation, glycolysis, fatty acid metabolism, peroxisome, mTORC1 signaling, reactive oxygen species pathways, and MYC targets—were enriched. Thus, CMV inverts the gestational trajectory observed under mock and HIV conditions, with antiviral programs becoming relatively stronger at term and metabolic remodeling predominating at early/mid-gestation. Consistent with a shared gestational imprint, all early/mid-gestation versus term contrasts exhibited enrichment of epithelial–mesenchymal transition, hypoxia, and angiogenesis pathways relative to term, regardless of exposure condition.

When virus-exposed HCs were compared with their gestational age-matched mock-exposed counterparts at early/mid-gestation, HIV and CMV elicited sharply divergent pathway-level responses (**Figure 3B**). HIV exposure at early/mid-gestation was associated with robust activation of interferon and inflammatory programs, including IFN-α and IFN-γ responses, TNFα–NFκB, IL2–STAT5, IL6–JAK–STAT3, inflammatory response, complement, and allograft rejection, together with induction of growth- and proliferation-related pathways such as MYC targets v1/v2 and epithelial–mesenchymal transition (EMT). Although few individual genes met the DEG threshold at this stage, pathway-level analysis revealed a coordinated, IFN-driven activation program in response to HIV exposure. In contrast, CMV exposure at early/mid-gestation did not induce classical interferon programs. IFN-α response and TNFα–NFκB were modestly downregulated, and other antiviral Hallmark pathways were not significantly enriched (**Figure 3B**). Instead, CMV primarily modulated stress- and remodeling-associated pathways, with downregulation of EMT, hypoxia, and reactive oxygen species pathways, and upregulation of MYC targets and allograft rejection.

At term, both viruses strongly engaged interferon pathways (**Figure 3C**). Term CMV-exposed versus term mock-exposed and term HIV-exposed versus term mock-exposed HCs each showed strong positive enrichment of IFN-α and IFN-γ responses, consistent with the shared antiviral core identified by overlapping DEGs. Beyond this common axis, term gestation CMV exposure was associated with coordinated repression of mitochondrial and lipid metabolic programs, including oxidative phosphorylation, glycolysis, fatty acid metabolism, peroxisome, mTORC1 signaling, and MYC targets v1/v2, together with reduced activity of related biosynthetic pathways. In contrast, term HIV-exposed HCs maintained or further amplified antiviral, inflammatory, and proliferative signatures, with positive enrichment of MYC targets v1/v2, E2F targets, G2M checkpoint, and IL6–JAK–STAT3 signaling; DNA repair was the only pathway showing negative enrichment. The direct term CMV versus term HIV analysis further clarified this divergence, showing that term CMV-exposed HCs were relatively enriched for interferon and inflammatory programs, whereas term HIV-exposed HCs retained stronger metabolic, biosynthetic, mTOR/MYC, and remodeling-associated pathway activity. Together, these analyses indicate that gestational stage establishes a dominant transcriptional baseline in HCs, with early/mid-gestation cells enriched for interferon and inflammatory programs and term cells enriched for metabolic pathways. Viral identity then modulates this baseline, with HIV largely preserving the gestational trajectory and CMV altering it by shifting the relative balance of interferon and metabolic programs across gestation (**Figure 3**).

### 2.6 CMV selectively perturbs placenta-enriched proteins at term-gestation

To relate transcriptional changes to the placental proteome, we intersected differentially expressed genes (DEGs) with Human Protein Atlas (HPA) annotations of placenta-enhanced and placenta-enriched proteins and visualized their distribution across contrasts using a three-layer Sankey diagram (**Figure 3D**). In HPA nomenclature, tissue-enriched genes show ≥5-fold higher expression in placenta relative to all other tissues, group-enriched genes are ≥5-fold higher in placenta and a limited set of related tissues, and tissue-enhanced genes display elevated, but not exclusive, placental expression.

Across all contrasts, 44 DEGs encoded placenta-enhanced or placenta-enriched proteins. Strikingly, most placenta-enhanced perturbations occurred in the term CMV versus term mock comparison, which showed broad down-regulation of extracellular matrix, adhesion, and myeloid receptor genes (e.g*., COL15A1, SPARC, VCAN, LYVE1, CSF1R, CSF2RA, IL12RB2*, and *TLR7*), together with up-regulation of a smaller set of interferon-associated enzymes and transcriptional regulators (e.g., *IDO1, IDO2, HPGDS, WARS1, EPAS1, FOXO4*, and *NFE2L3*).

A partially overlapping subset of these placenta-associated genes also differed in the CMV gestational contrast (early/mid versus term), suggesting that the transcriptional programs affected by CMV at term intersect with gestational remodeling of placental structural and immune pathways. In contrast, term HIV-exposed HCs compared with mock controls perturbed only a single placenta-enhanced gene (*WARS1*), indicating that CMV exerts a markedly stronger placenta-specific transcriptional impact at term.

### 2.7 Targeted reactome analysis reveals CMV-specific perturbation of WNT signaling

**Figure 4:**
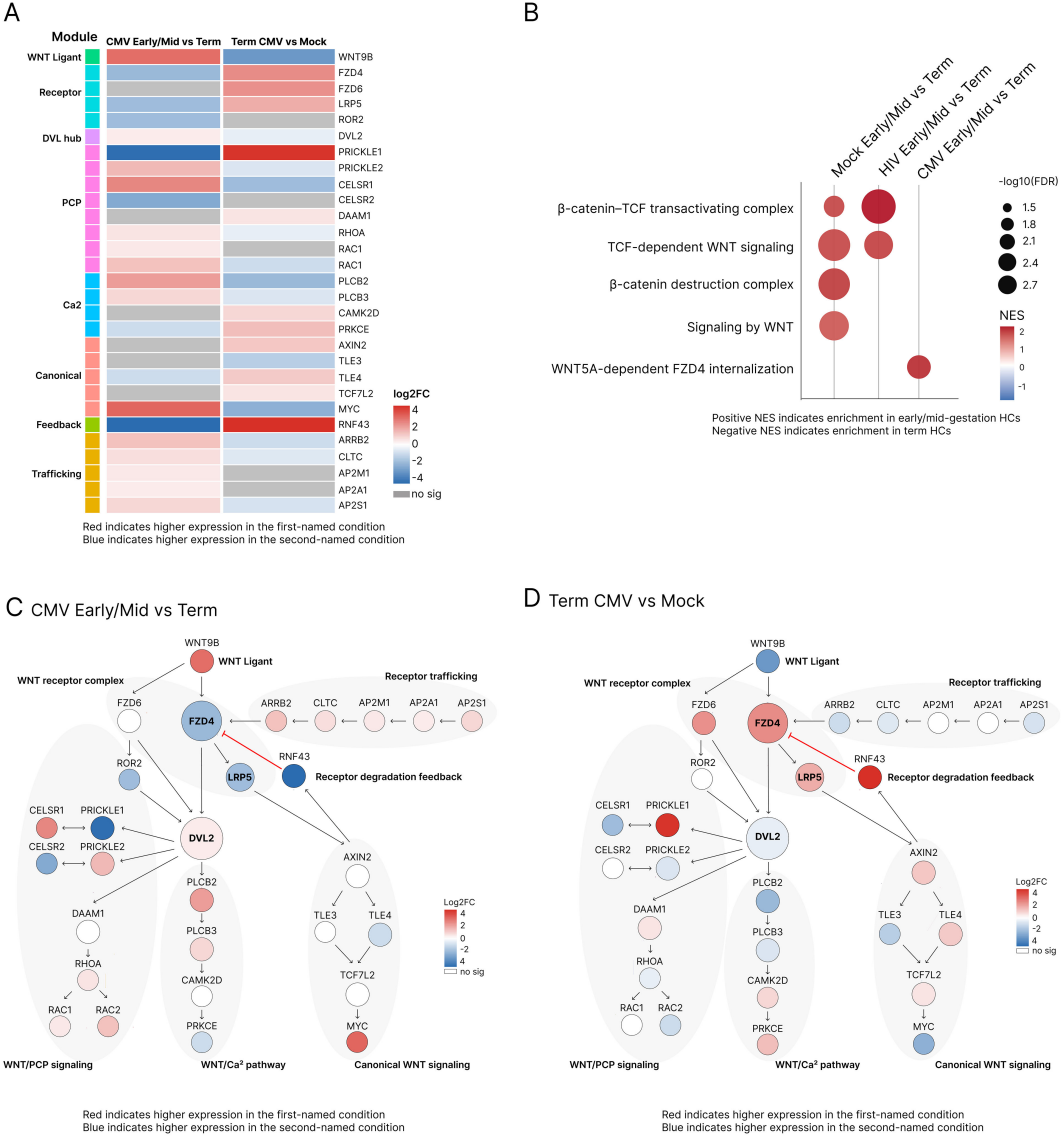
Gestational-stage– and virus-specific modulation of WNT signaling pathways in Hofbauer cells. **(A)** Heatmap showing log_2_ fold-change of selected WNT pathway genes contributing to enriched Reactome WNT pathways. Genes are grouped by functional annotation, including WNT ligands, receptors, DVL hub components, PCP/non-canonical signaling, Ca^2+^-associated signaling, canonical β-catenin signaling, receptor-feedback regulation, and trafficking. Red indicates higher expression in the first-named condition, blue indicates higher expression in the second-named condition, and grey indicates genes that did not meet the significance threshold in the indicated contrast. **(B)** Dot plot summarizing Reactome WNT pathway enrichment across gestational-stage contrasts comparing early/mid-gestation and term HCs under mock-exposed, HIV-exposed, and CMV-exposed conditions. Only pathways significant in at least one contrast are shown (FDR ≤ 0.05). For contrasts labeled early/mid versus term, positive NES values indicate enrichment in early/mid-gestation HCs, whereas negative NES values indicate enrichment in term HCs. Dot color indicates NES and dot size reflects statistical significance, shown as −log_10_(FDR). **(C)** Network representation of WNT signaling components differentially expressed between early/mid-gestation and term CMV-exposed HCs. Nodes represent WNT pathway genes and edges indicate curated interactions within the Reactome WNT signaling network. Node color indicates log_2_ fold-change for the early/mid CMV versus term CMV comparison; red indicates higher expression in early/mid CMV-exposed HCs, blue indicates higher expression in term CMV-exposed HCs, and white/grey indicates genes that did not meet the significance threshold. **(D)** Network representation of WNT signaling components differentially expressed in term CMV-exposed versus term mock-exposed HCs. Nodes represent WNT pathway genes and edges represent curated pathway interactions. Node color indicates log_2_ fold-change for the term CMV versus term mock comparison; red indicates higher expression in term CMV-exposed HCs, blue indicates higher expression in term mock-exposed HCs, and white/grey indicates genes that did not meet the significance threshold.

Given the convergence of extracellular matrix remodeling, metabolic perturbations, and placenta-specific transcriptional signatures observed in term CMV-exposed HCs, we next examined WNT-related genes and Reactome WNT pathways across gestation-stage and virus exposure contrasts. Gene-level analysis of selected WNT pathway components revealed contrast-dependent regulation of receptors and intracellular signaling mediators, including FZD4, FZD6, LRP5, DVL2, and RNF43 (**Figure 4A**). Reactome pathway enrichment analysis showed selective enrichment of distinct WNT signaling modules across gestational stages and exposure contexts (**Figure 4B**). For all gestational-stage contrasts shown in **Figure 4B**, positive NES values indicate enrichment in the first-named condition, early/mid-gestation HCs, whereas negative NES values indicate enrichment in term HCs.

In the mock and HIV gestational contrasts, canonical WNT/β-catenin-related pathways, including TCF-dependent signaling in response to WNT and formation of the β-catenin–TCF transactivating complex, were enriched in early/mid-gestation HCs relative to term HCs (**Figure 4B**). In contrast, WNT5A-dependent internalization of FZD4, a non-canonical WNT module associated with receptor trafficking and signal routing, was specifically enriched in the CMV early/mid versus CMV term comparison (NES = 1.9, FDR < 0.05; **Figure 4B**). Thus, CMV exposure was associated with a distinct gestational shift toward non-canonical receptor-trafficking–related WNT pathway enrichment, rather than the canonical WNT/β-catenin pattern observed in mock and HIV gestational comparisons.

Network reconstruction revealed that WNT-associated transcriptional changes were organized into distinct signaling modules corresponding to canonical and non-canonical branches of the pathway (**Figures 4C, D**). In the CMV early/mid-gestation versus the CMV term comparison, differential expression was distributed across multiple downstream WNT signaling components, including planar cell polarity (PCP) associated genes (*CELSR1, CELSR2, PRICKLE1*, *PRICKLE2)*, cytoskeletal regulators (*RHOA, RAC1*, *RAC2)*, WNT/Ca^2+^ signaling genes (*PLCB2*, *PLCB3)*, and the central pathway integrator *DVL2 (***Figure 4C***)*. In contrast, the term CMV versus mock network showed a receptor-centered pattern, with increased expression of FZD4, FZD6, LRP5, and the WNT negative regulator RNF43 (**Figure 4D**). Consistent with this receptor- and feedback-associated architecture, most downstream canonical and non-canonical WNT components did not exhibit significant differential expression in the term CMV versus mock comparison.

Because these analyses were performed in isolated HCs, WNT-related changes should be interpreted as HC-associated transcriptional changes rather than direct evidence of altered trophoblast WNT signaling or placental tissue remodeling. In this context, WNT pathway alterations may reflect changes in macrophage programs involved in cell–cell communication, inflammatory regulation, extracellular matrix interaction, receptor trafficking, and responsiveness to developmental cues within the villous microenvironment. Thus, the WNT findings provide an HC-centered link between viral exposure and pathways relevant to maternal–fetal interface biology, but their tissue-level consequences will require validation in intact placental models.

### 2.8 Co-expression modules capture stage-by-virus interactions in immune–metabolic networks

**Figure 5:**
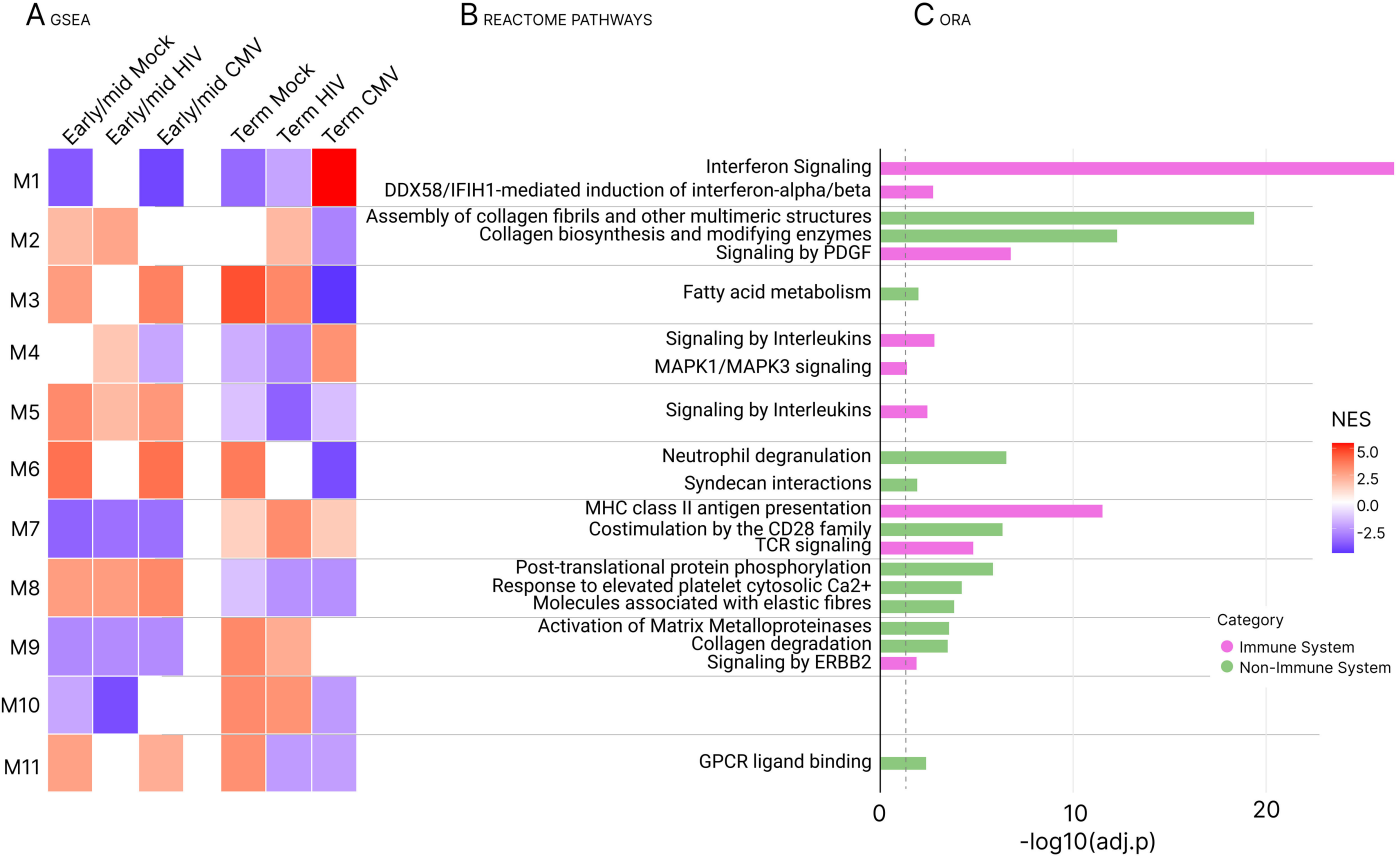
Co-expression modules reveal coordinated immune–metabolic programs across gestation and in response to viral stimuli. **(A)** Normalized enrichment scores (NES) from module-level GSEA across six experimental groups (early/mid vs term × mock-exposed, HIV, CMV). Each row represents a CEMiTool module (M1–M11), and colors indicate direction and strength of enrichment. **(B)** Reactome over-representation analysis of CEMiTool module gene sets. Labels summarize the leading Reactome pathway annotations for each module and are based on gene-set overlap; they should not be interpreted as evidence for the presence or activity of non-HC cell types such as T cells, neutrophils, or fibroblasts. **(C)** Reactome over-representation analysis (ORA) results for selected module gene sets. Bars represent –log10(adjusted p), with color indicating whether the pathway is primarily immune-related (purple) or non-immune cellular/metabolic (green).

We next examined how transcripts co-vary across conditions. Using CEMiTool, we identified 11 co-expression modules (M1–M11) representing coherent biological programs with distinct Reactome enrichments (**Figures 5A–C**). M1 comprised a classic IFN/innate antiviral program, including interferon signaling and DDX58/IFIH1-mediated induction of type I interferon. M2 was enriched for extracellular matrix and stromal-remodeling pathway annotations, including collagen biosynthesis and PDGF signaling, whereas M3 included fatty acid metabolism together with myeloid vesicle/degranulation-related Reactome annotations. Modules M4–M6 represented interleukin/TNF/MAPK and inflammatory pathway annotations. M7 included antigen-presentation and T-cell interaction/costimulatory pathway annotations, including MHC class II antigen presentation, CD28-family co-stimulation, and interferon signaling. M8, M9, and M11 were enriched for platelet activation, ECM remodeling, and GPCR/lipoprotein pathway annotations. Because these Reactome terms are derived from gene-set annotations, they should be interpreted as pathway labels reflecting shared transcriptional programs in HCs rather than as evidence for the presence or activity of non-HC cell types such as T cells, neutrophils, or fibroblasts.

We then evaluated module behavior across the six HC classes: early/mid-gestation mock-exposed, early/mid-gestation HIV-exposed, early/mid-gestation CMV-exposed, term mock-exposed, term HIV-exposed, and term CMV-exposed. Module-level enrichment analysis revealed a striking skew for the IFN module M1. Term CMV-exposed HCs showed very strong positive enrichment of M1 (NES ≈ +5.4), whereas all other groups showed negative enrichment (**Figure 5C**).

The fatty-acid metabolism module M3 showed the opposite trend relative to term CMV-exposed cells. Instead of being activated, M3 was strongly suppressed in term CMV-exposed HCs (NES ≈ −4.1), whereas all other conditions showed positive enrichment, suggesting a CMV-specific reduction in oxidative lipid metabolism at term. A similar pattern emerged for the collagen/PDGF module M2. Early/mid-gestation mock, HIV and CMV showed positive enrichment, consistent with active ECM-remodeling programs early in gestation, whereas term mock and CMV-exposed groups shifted M2 toward negative enrichment.

The antigen-presentation/T-cell interaction and costimulatory annotation module M7 displayed a different profile. Here, the largest positive shift was seen in term HIV-exposed HCs (NES ≈ +3.4), with term mock and term CMV-exposed groups showing more modest enrichment. In contrast, all three early/mid-gestation groups showed negative enrichment, indicating that MHC-II antigen-presentation and T-cell interaction/costimulatory pathway annotations are predominantly features of term HCs regardless of viral exposure.

Overall, these module-level patterns support a two-step model of HC regulation. Gestational age first establishes the baseline architecture of IFN, antigen-presentation and metabolic networks; viral exposure then rewires this scaffold in distinct ways. At term, CMV couples a large IFN upregulation (M1) with coordinated repression of lipid metabolic (M3) and ECM-related (M2) modules, whereas HIV predominantly amplifies the MHC-II antigen-presentation and T-cell interaction/costimulatory annotation module (M7).

### 2.9 Cytokine secretion mirrors transcript programs

**Figure 6:**
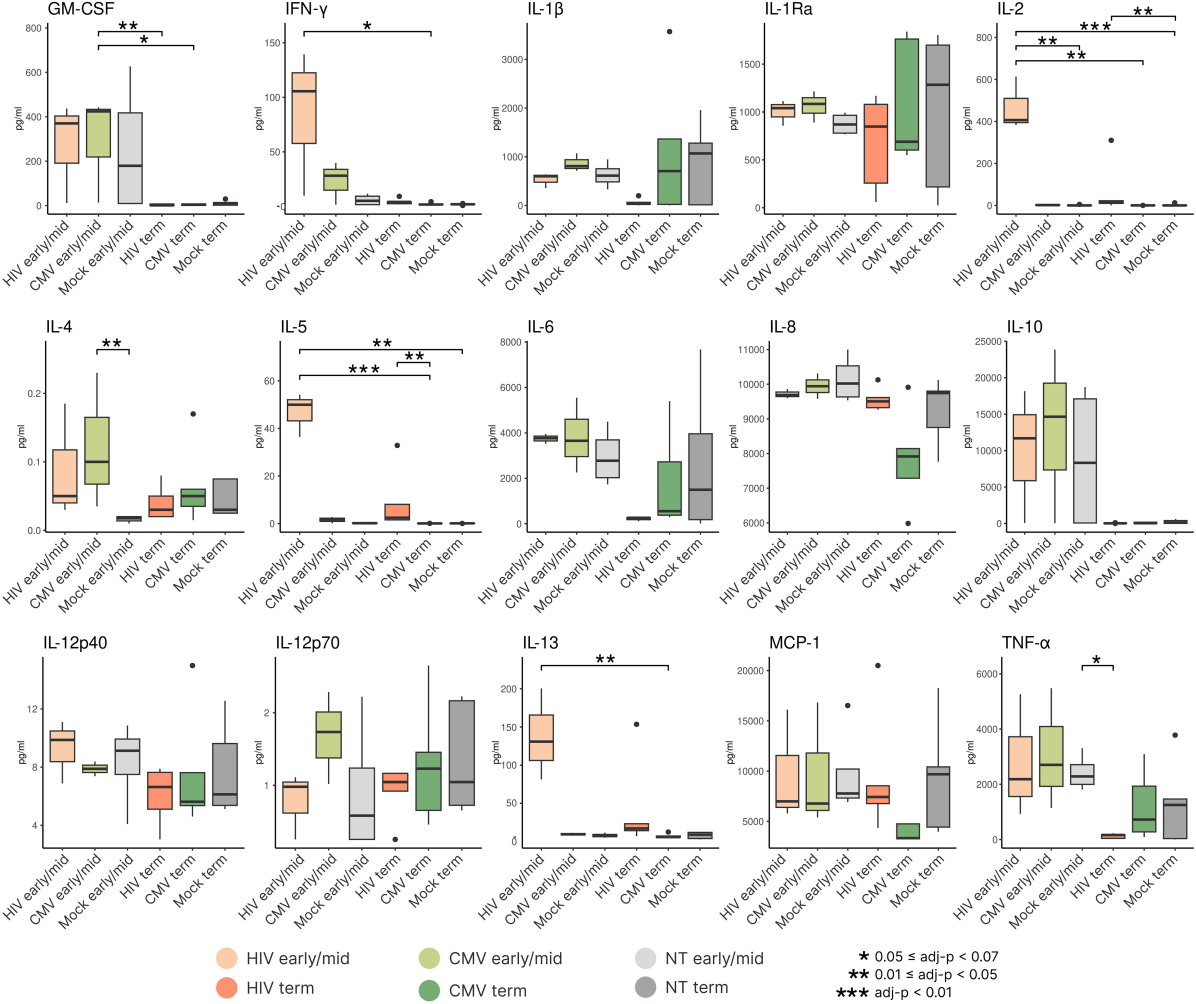
Cytokine concentrations in HC culture supernatants across gestational stage and viral exposure conditions. Multiplex bead assays quantified GM-CSF, IFN-γ, IL-1β, IL-1Ra, IL-2, IL-4, IL-5, IL-6, IL-8, IL-10, IL-12p40, IL-12p70, IL-13, MCP-1, and TNF-α in supernatants from early/mid-gestation and term-gestation HCs exposed to HIV, CMV, or mock conditions. Colors indicate group, and boxplots summarize cytokine concentrations in pg/mL. Boxes indicate the interquartile range, center lines indicate the median, whiskers indicate 1.5× the interquartile range, and points indicate outliers. Pairwise comparisons were performed using Dunn’s *post-hoc* test with Bonferroni correction after Kruskal–Wallis testing. Asterisks indicate adjusted p-values: * 0.05 ≤ adj-p < 0.07, ** adj-p < 0.05, and *** adj-p < 0.01.

At early/mid-gestation, cytokine output was generally higher than at term for several analytes, with HIV-exposed cultures showing the strongest antiviral/inflammatory cytokine profile (**Figure 6**). Early/mid-gestation HIV-exposed cultures showed the highest IFN-γ, IL-2, and IL-5 signals, with statistically supported pairwise differences for IL-2 and IL-5 against selected mock or term groups. GM-CSF was also numerically higher in early/mid HIV-exposed cultures, although within-group variability was substantial and the early/mid HIV versus mock comparison was not interpreted as a statistically supported increase. These cytokine patterns are consistent with the interferon- and inflammation-enriched Hallmark signatures observed in the early/mid HIV versus mock transcriptomic contrast. IL-13 also showed higher levels in early/mid HIV-exposed cultures relative to selected term groups, indicating that the soluble milieu includes both antiviral/inflammatory and immune-modulatory components rather than mapping cleanly onto a single “type 1” axis.

CMV exposure at early/mid-gestation produced a distinct cytokine profile. IL-4 was higher in early/mid CMV-exposed cultures than in early/mid mock-exposed cultures, consistent with the pairwise statistical annotation in **Figure 6**. In contrast, we did not interpret IL-5 as a CMV-specific increase because the strongest IL-5 signal was observed in early/mid HIV-exposed cultures. Together, these patterns align with the transcriptomic finding that early/mid CMV exposure does not strongly induce classical interferon Hallmark pathways and instead preferentially modulates stress- and remodeling-associated programs.

At term, cytokine concentrations were generally lower for several antiviral and lymphoid-associated cytokines, including IFN-γ, IL-2, IL-5, and IL-13. Differences among term exposure groups were more modest and variable. IL-1β and IL-1Ra showed broad variability and were not interpreted as statistically supported CMV-specific increases in the absence of pairwise annotations. TNF-α showed only a trend-level pairwise difference in the corrected analysis and was therefore interpreted cautiously rather than as a virus-specific term response. Overall, the Luminex profiles support the conclusion that gestational stage shapes baseline cytokine output, while viral identity modulates selected cytokine responses.

## 3 Discussion

The placenta is a rapidly changing, highly specialized organ that must simultaneously support fetal growth, maintain maternal–fetal tolerance, and provide barrier and immune protection over a relatively short developmental window (1, 6). HCs, the fetal macrophages of the villous core, are central to these demands, integrating environmental, developmental, and pathogen-derived signals throughout gestation (8–13, 21). In this study, we used primary HCs from early/mid-gestation and term placentas to examine how gestational age and viral identity (HIV and CMV) interact to shape innate immune programs at the maternal–fetal interface. By integrating differential gene expression, pathway analysis, co-expression modules, and cytokine profiling, we identify a stage-by-virus interaction that reveals how developmental state conditions antiviral responses in HCs.

Across mock-exposed cultures, the gestational stage emerged as a dominant organizing axis of HC biology. Early/mid-gestation HCs were characterized by interferon- and inflammation-enriched transcriptional programs coupled to relatively low metabolic activity, whereas term HCs exhibited a coordinated shift toward oxidative phosphorylation and lipid metabolism with attenuation of interferon signatures. This developmental transition was largely preserved in HIV-exposed cells, indicating that gestational programming establishes a robust immune–metabolic scaffold upon which viral stimuli act rather than redefining it *de novo*.

Within this gestational framework, viral identity dictated how the developmental scaffold was engaged. HIV largely exploited pre-existing interferon-primed circuitry, particularly at early/mid-gestation, and at term overlaid strong interferon, inflammatory, and cell-cycle programs without broadly repressing metabolic pathways. In contrast, CMV partially rewired this gestational imprint, shifting the balance of interferon and metabolic programs across gestation. At term, CMV coupled robust interferon activation to coordinated suppression of mitochondrial and lipid metabolic pathways and related biosynthetic modules, consistent with a transcriptional state combining strong antiviral signaling with coordinated repression of metabolic programs. Together, these findings suggest that gestational age not only shapes baseline placental macrophage function but also determines how viral exposures are translated into immune and metabolic outcomes.

### 3.1 Developmental programming of Hofbauer cells across gestation

Our data reinforce and refine the concept that placental immunity is developmentally programmed. Prior work has shown that HCs emerge early in gestation, display predominantly regulatory, M2-like phenotypes, and progressively adapt to balance tissue growth, immune tolerance, and host defense across pregnancy (8–12). Swieboda et al. demonstrated that early/mid-gestation HCs mount stronger ligand-driven antiviral responses than term HCs, including heightened responsiveness to RIG-I agonists and IFN-γ (8). Johnson et al. similarly reported robust early gestation antiviral states that may limit *in utero* HIV transmission (13, 14, 21).

In line with these observations, our Hallmark pathway analyses revealed a clear gestational imprint in mock-exposed cultures. Early/mid-gestation HCs were enriched for interferon and inflammatory programs, including IFN-α and IFN-γ responses, TNFα–NFκB signaling, IL2–STAT5, IL6–JAK–STAT3, complement, and allograft rejection pathways. In contrast, term HCs preferentially upregulated metabolic and growth-associated pathways, including oxidative phosphorylation, glycolysis, fatty acid metabolism, mTORC1 signaling, and MYC target programs. Together, these data support a developmental transition from an ‘interferon-primed, metabolism-low’ baseline early in gestation to a more ‘metabolism-high, growth-oriented’ state at term. It is consistent with the evolving functional demands of the placenta as it undergoes vascular expansion, villous remodeling, and preparation for parturition (1, 3–6).

CMV, however, diverges markedly from the canonical gestational trajectory observed in mock-exposed cultures. In CMV-exposed HCs, comparison of early/mid-gestation versus term revealed downregulation of both IFN-γ and IFN-α signaling programs at early/mid-gestation, suggesting relative attenuation of interferon responses early in gestation (**Figure 3A**). This pattern is consistent with extensive evidence that CMV has evolved multiple mechanisms to antagonize type I interferon signaling (25–27). In particular, CMV can reduce the accumulation and phosphorylation of IFN signaling kinases such as *JAK1* and *STAT2*, thereby impairing downstream type I interferon responses, and has also been shown to directly interfere with *STAT2* expression and signaling (24).

In parallel, CMV-exposed HCs displayed enrichment of multiple metabolic and biosynthetic pathways at early/mid-gestation relative to term, including oxidative phosphorylation, glycolysis, fatty acid metabolism, peroxisome function, reactive oxygen species pathways, hypoxia, and MYC target programs. These metabolic signatures emerged in the absence of strong enrichment for classical interferon pathways in early/mid-gestation CMV versus mock comparisons, suggesting that CMV does not simply engage the default interferon-primed antiviral wiring of HCs. Instead, in contrast to mock-exposed cultures, CMV appears to favor a stress- and remodeling-oriented state early in gestation, characterized by enhanced mitochondrial and lipid metabolic capacity rather than canonical IFN signaling.

This pattern is consistent with prior studies showing that CMV actively rewires host-cell metabolism to support viral replication, including coordinated upregulation of glycolysis and mitochondrial oxidative phosphorylation, increased mitochondrial transcription and translation capacity, and enhanced lipid biosynthesis required for viral assembly and envelopment (28). Systems-level analyses further indicate that CMV temporally decouples metabolic activation from antiviral effector programs, thereby delaying robust interferon engagement while exploiting host metabolic plasticity (29). In our data, this early metabolic bias precedes a marked shift at term, where CMV exposure couples strong interferon activation with coordinated repression of mitochondrial and lipid metabolic pathways, consistent with an energy-restricted yet highly antiviral state (28).

These stage-specific patterns have important clinical implications. Epidemiological studies indicate that the risk of *in utero* viral transmission increases with advancing gestational age, whereas early infections, although less frequently transmitted, are disproportionately associated with severe fetal outcomes (15–19). Our findings suggest that, at the cellular level, these epidemiological patterns may reflect a shifting balance between antiviral pressure, metabolic demands, and placental structural integrity across gestation.

### 3.2 Term gestation as a vulnerability window: shared antiviral core, divergent downstream transcriptional programs

At term, both HIV and CMV induced a shared interferon-rich antiviral signature, comprising 85 overlapping DEGs. This gene set included canonical IFN-stimulated genes, antigen-processing machinery, chemokines and cytokines, and restriction/adapter proteins. Reactome enrichment confirmed strong representation of type I and type II interferon signaling, antigen processing and cross-presentation, and interleukin-12/27/20 family pathways. These data indicate that, despite major differences in life cycle and tropism, HIV and CMV converge on a similar antiviral backbone in term HCs. This convergence is consistent with prior work showing that HCs can sense and restrict HIV, upregulate interferon-stimulated genes, and sequester virions in specialized compartments accessible to neutralizing antibodies (15, 16, 22). The presence of a similar IFN/MHC core in CMV-exposed HCs aligns with broader work on placental antiviral defense, in which trophoblasts and macrophages activate type I/III interferons and antigen-presentation pathways in response to viral challenge (2, 13, 21, 22, 24).

However, the shared interferon core is only part of the story. Our Hallmark and co-expression analyses show that CMV and HIV engage distinct downstream transcriptional programs at term, a pattern further supported by a direct comparison of term CMV and term HIV. This direct contrast showed that term CMV-exposed HCs were relatively enriched for interferon alpha/beta, interferon gamma, DDX58/IFIH1-mediated interferon induction, TRAF6/IRF7 activation, TNFα–NFκB signaling, and inflammatory response pathways. In contrast, term HIV-exposed HCs were relatively enriched for oxidative phosphorylation, glycolysis, fatty-acid metabolism, peroxisome, mTORC1 signaling, MYC targets, PI3K–AKT–mTOR signaling, translation, amino acid–stress responses, and epithelial–mesenchymal transition. These findings refine our interpretation by showing that term CMV-exposed HCs are not simply a stronger version of the shared antiviral response. Term CMV-exposed HCs are shifted toward an IFN-dominant inflammatory state with relative reduction of metabolic, biosynthetic, and remodeling-associated transcriptional programs, whereas term HIV-exposed HCs retain comparatively stronger metabolic and growth-associated activity. Thus, while HIV and CMV share a common antiviral backbone at term, CMV couples this response to broader transcriptional remodeling, whereas HIV preserves relatively stronger metabolic, translational and growth-related programs.

#### 3.2.1 WNT signaling as a contextual regulator of immune–metabolic–structural programs

WNT pathways are increasingly recognized as regulators linking immune activation, cellular metabolism, and tissue architecture, particularly in developmental tissues and tissue-resident macrophages (30–33). Given that term CMV uniquely combined robust interferon activation with coordinated suppression of metabolic and ECM/junctional programs, we examined whether WNT signaling also differed across gestation. Reactome pathway analysis of early/mid-gestation versus term comparisons revealed that WNT signaling behaved differently across viral conditions: mock and HIV contrasts showed broadly similar patterns, whereas CMV exposure uniquely enriched the non-canonical WNT5A-dependent internalization of *FZD4* pathway.

At early/mid gestation, CMV-associated WNT changes were distributed across multiple downstream signaling branches, including planar cell polarity components, cytoskeletal regulators, and elements of the WNT/Ca^2+^ pathway. Canonical pathway outputs were also represented, indicating that early/mid gestation differences were not restricted to a single non-canonical branch but instead spanned several downstream WNT modules. This pattern is consistent with broad remodeling of pathway architecture at the level of signal routing and downstream effector engagement.

In contrast, term CMV exposure revealed a markedly different WNT network architecture, characterized primarily by increased expression of *FZD4, FZD6*, the co-receptor *LRP5*, and the transmembrane E3 ubiquitin ligase *RNF43*. *RNF43* negatively regulates WNT signaling by promoting ubiquitination, endocytosis, and degradation of Frizzled receptors, thereby restricting pathway activation (34). The predominance of receptor- and feedback-associated changes, together with limited differential expression across many downstream canonical and non-canonical components, suggests that CMV exposure at term primarily alters WNT pathway responsiveness through receptor availability and turnover.

This interpretation is consistent with prior work showing that CMV perturbs both canonical and non-canonical WNT regulatory networks. CMV has been shown to dysregulate canonical WNT/β-catenin signaling in placental extravillous trophoblasts (35) and to modulate expression of the non-canonical receptor *ROR2* in a manner that alters trophoblast migration (36). Pharmacologic perturbation of WNT-related signaling can influence CMV replication, indicating that CMV is functionally sensitive to the state of the WNT pathway (37). Together, these observations suggest that CMV interacts with WNT signaling across multiple regulatory levels, including receptor availability, downstream transcriptional outputs, and cellular functional responses. In this context, WNT signaling appears to function not as a primary driver of CMV-induced transcriptional changes, but rather as a regulatory layer that accompanies CMV-driven immune, metabolic, and structural shifts in Hofbauer cells.

Although WNT signaling is often discussed in trophoblast and placental development, macrophages can also act as both sources and recipients of WNT signals and use WNT pathways to regulate inflammatory tone, angiogenesis, tissue repair, and remodeling-associated responses (33, 38–41). Given that HCs are fetal villous macrophages implicated in placental immune regulation, angiogenesis, and responses to infection or inflammation (42), the WNT-related changes observed here may reflect HC regulatory programs relevant to maternal–fetal interface biology, but their tissue-level consequences require validation in intact placental models.

### 3.3 Term CMV signatures link antiviral activation to placental structure and vascular remodeling

A central aspect of this work is the integration of DEG data with HPA annotations to focus on placenta-specific proteins (43, 44). Using this approach, we found that CMV exposure at term altered 34 placenta-enhanced/tissue-enriched genes, compared with only one gene (*WARS1*) in the matched term HIV versus mock contrast. Term CMV exposure prominently downregulated genes encoding ECM and adhesion molecules, as well as several immune receptors relevant to macrophage–trophoblast and leukocyte crosstalk, while upregulating a smaller set of interferon-inducible genes and transcriptional regulators.

These findings resonate with the placental pathology literature, which describes ECM disorganization, altered vascular remodeling, and junctional defects in conditions such as fetal growth restriction and preeclampsia (3–5, 45). For example, VE-cadherin–dependent trophoblast invasion and spiral artery remodeling are essential for appropriate uteroplacental blood flow, and perturbations in endothelial–trophoblast/ECM complexes can compromise perfusion and barrier function (45). The coordinated downregulation of collagen- and matrix-related genes in the CEMiTool module 2, together with suppression of metabolic pathways supporting biosynthesis and energy production, suggests disruption of macrophage programs that sustain extracellular matrix organization and stromal homeostasis within the placental villous microenvironment, consistent with a structurally and metabolically stressed tissue state.

Taken together, these findings suggest that CMV exposure at term drives HCs toward an “IFN-on, metabolism/ECM-off” transcriptional state that may compromise villous integrity, vascular remodeling, and barrier function. This interpretation is consistent with clinical observations linking congenital CMV infection to fetal growth restriction, placental lesions, and adverse pregnancy outcomes (3–5, 17, 18, 23), and with our prior work showing that CMV enhances placental susceptibility to HIV infection and replication (24).

### 3.4 Mechanistic links to CMV-mediated enhancement of HIV susceptibility

Our findings provide a mechanistic framework for understanding how CMV exposure may enhance HIV susceptibility at the maternal–fetal interface. Johnson et al. reported that CMV increases HIV replication in HCs, upregulates *CCR5*, and shifts cytokine profiles toward a more pro-inflammatory state with reduced IL-10 (24). Other work has highlighted *CCR5* as a key therapeutic target in HIV infection (46) and emphasized the importance of local chemokine gradients and tissue architecture in regulating viral entry and spread (2, 13, 46, 47).

In term HCs, we observe that CMV and HIV both engage the IFN antiviral core, but only CMV drives deep metabolic and ECM/junctional repression and widespread alterations in placenta-enriched genes. This CMV-specific configuration is also consistent with the WNT remodeling described above, where receptor-level regulation and feedback control were preferentially observed at term. Notably, WNT/β-catenin signaling has been shown to restrict HIV replication and transcription in several cellular systems, including monocytes/macrophages and astrocytes (48, 49). CMV-associated perturbation of WNT signaling in term HCs may therefore reduce β-catenin–mediated restriction pathways and contribute to a more permissive cellular state for HIV infection. In contrast to the strong transcriptomic divergence observed at term, cytokine differences among term exposure groups were modest and variable, indicating that the proposed CMV-mediated enhancement of HIV permissiveness is supported primarily by transcriptional remodeling of interferon, metabolic, ECM/junctional, and WNT-associated programs rather than by a term CMV-specific cytokine signature. Taken together, these findings support a working model in which CMV exposure at term drives HCs toward an antiviral yet structurally and metabolically constrained state. CMV-induced repression of ECM/collagen and junctional pathways, together with suppression of mitochondrial and lipid metabolism and perturbation of placenta-enriched genes, may weaken villous structural integrity and disrupt trophoblast–endothelial interactions (3–5, 44). In parallel, CMV-associated remodeling of WNT signaling could further influence cellular permissiveness, as β-catenin activity has been shown to restrict HIV replication and transcription in several cell types. Superimposed on this stressed microenvironment, IFN-dominant inflammatory signaling together with altered chemokine and tissue-remodeling programs may maintain immune niches that favor recruitment and activation of CCR5⁺ HIV target cells (24, 45). In this scenario, CMV simultaneously alters structural integrity, metabolic capacity, and signaling pathways that normally limit viral replication, thereby creating conditions that may enhance HIV susceptibility at the maternal–fetal interface. Importantly, because our *in vitro* system focuses on isolated HCs, these mechanisms should be considered hypotheses generated from macrophage-centered data rather than definitive causal pathways within the intact placental microenvironment.

### 3.5 Early/mid-gestation: distinct HIV and CMV programs in a highly dynamic organ

At early/mid-gestation, few individual genes met DEG thresholds, but pathway/module analyses together with cytokine profiling revealed clear qualitative differences between HIV and CMV responses. HIV-exposed early/mid-gestation HCs showed enrichment of interferon, inflammatory, and cell-cycle programs and displayed the highest IFN-γ, IL-2, and IL-5 cytokine signals, with GM-CSF showing a numerical increase but not a statistically supported early/mid HIV versus mock difference. These findings align with prior work from our group showing robust innate antiviral responsiveness to HIV and RIG-I agonists in first- and second-trimester HCs (8, 13, 14).

In contrast, CMV-exposed early/mid-gestation HCs did not show positive enrichment for classical interferon Hallmarks and instead modulated EMT, hypoxia, reactive oxygen species, and MYC programs, together with higher IL-4 relative to early/mid mock-exposed cultures. IL-5 was not interpreted as CMV-specific because the strongest IL-5 signal was observed in early/mid HIV-exposed cultures. This pattern supports a more immune-modulatory, remodeling-oriented response, compatible with the need to maintain tolerance and ongoing villous development in a rapidly changing organ (2, 6, 11, 12, 50). It also suggests that CMV can establish a foothold in HCs without triggering strong canonical interferon activation early in gestation, which may help explain the severe—but sometimes clinically silent—placental sequelae of early CMV infection (17, 18, 23).

### 3.6 Methodological considerations, limitations and future directions

Several limitations should be acknowledged. First, the number of early- and mid-gestation placentas is limited, reflecting both ethical and logistical constraints on obtaining healthy material at these stages and the impact of recent legal restrictions on tissue procurement. This reduces the power to detect subtle stage-specific differences and may underestimate the diversity of HC states *in vivo*. Nonetheless, the consistency of our findings across donors, analytic layers (DEGs, pathways, modules), and independent readouts (cytokines) supports the robustness of the main patterns.

Second, our experiments focus on purified HCs and short-term *in vitro* exposures. This design allows precise control and a clean readout of HC-intrinsic programs, but it does not fully recapitulate the placental multicellular network, including trophoblasts, endothelial cells, decidual immune cells, and maternal blood components (2, 6, 47). In addition, mock-exposed cultures were medium-only controls and did not include virus-free matched producer-cell supernatants. Thus, potential producer-cell-derived components in viral stocks, particularly HCMV TB40/E propagated in ARPE-19 cells, cannot be fully excluded.

Third, HIV-1 BaL is a laboratory-adapted macrophage-tropic strain and does not capture the full genetic diversity of contemporary clinical or transmitted/founder HIV isolates. Future studies using clinical isolates will be important for validation. In addition, gravidity status was not available in the clinical metadata for this study, preventing stratification by primigravid versus multigravid status.

Future studies using matched virus-free producer-cell supernatant controls, contemporary clinical viral isolates, organoid systems, ex vivo perfusion, spatial transcriptomic approaches and more comprehensive clinical metadata could help validate how the HC programs we describe are integrated at the tissue level. Despite these caveats, our study provides a coherent, biologically grounded framework in which developmental stage sets the baseline wiring of HC antiviral and metabolic networks, and viral identity determines whether that wiring is associated with preservation or disruption of immune, metabolic, and remodeling-associated transcriptional programs.

## 4 Conclusions

Gestational stage is a major determinant of HC biology, establishing a developmental immune–metabolic baseline upon which viral exposures act. At term, HIV and CMV converged on a shared interferon-rich antiviral program characterized by JAK–STAT activation, induction of antiviral effectors, and enhanced antigen-presentation machinery. However, the downstream consequences of this shared antiviral core differed markedly between viruses. CMV-exposed term HCs were relatively enriched for interferon alpha/beta, interferon gamma, TNFα–NFκB, and inflammatory pathways, whereas HIV-exposed term HCs retained comparatively stronger metabolic, translational, mTOR/MYC, lipid metabolism, glycolytic, and remodeling-associated transcriptional programs. These findings support a model in which CMV shifts term HCs toward an IFN-dominant and relatively metabolism/remodeling-low transcriptional state, whereas HIV preserves a more biosynthetic and metabolically active profile.

At early/mid gestation, HIV and CMV also diverged at the pathway level despite minimal gene-level differential expression. HIV induced coordinated interferon- and inflammation-associated pathway activity, whereas CMV preferentially modulated remodeling-, stress-, and immune-modulatory programs and produced a distinct cytokine profile. Together, these findings support a model in which gestational programming shapes how HCs translate viral exposure into immune, metabolic, and transcriptional programs. Within this framework, CMV emerges as the virus that most strongly rewires term HCs toward an antiviral but metabolically and structurally constrained state, providing a plausible HC-centered mechanism by which CMV could contribute to placental vulnerability and HIV permissiveness at the maternal–fetal interface. More broadly, these data link placental antiviral defense to tissue metabolic and structural integrity and support future studies aimed at preserving placental resilience during late gestation.

## 5 Materials and methods

### 5.1 Placental collection and tissue processing

Placental collections were approved by the Emory University Institutional Review Board (IRB 000217715), and written informed consent was obtained from all participants. Membrane-free villous placental tissue was collected immediately after delivery and processed as previously described (12). Briefly, placental tissue was thoroughly washed and mechanically dispersed in Hank’s balanced salt solution (HBSS) to minimize peripheral blood contamination. Minced tissue was digested in complete medium containing 0.2% trypsin/EDTA (Sigma-Aldrich, St. Louis, MO, USA) for 1 hour, followed by digestion with collagenase A (1 mg/mL; Worthington Biochemical, Lakewood, NJ, USA) and DNase I (0.2 mg/mL; Sigma-Aldrich) for 1 hour at 37 °C in a shaking water bath. The digested tissue was washed with PBS and passed through gauze and a 70 μm cell strainer (BD-Falcon Biosciences, Lexington, TN, USA). The final RNA-seq dataset included 23 independent donor-derived HC RNA-seq samples. This included 8 early/mid-gestation samples and 15 term samples, distributed across six experimental groups: early/mid mock-exposed HCs (n = 3), early/mid HIV-exposed HCs (n = 3), early/mid CMV-exposed HCs (n = 2), term mock-exposed HCs (n = 5), term HIV-exposed HCs (n = 5), and term CMV-exposed HCs (n = 5). Biological replicates corresponded to independent donor-derived HC samples, and sample-level metadata are provided in **Supplementary Table 1**.

### 5.2 Isolation and culture of Hofbauer cells

The mononuclear cell fraction was isolated by density gradient centrifugation on Histopaque-1077 (Sigma-Aldrich). Hofbauer cells were purified by CD14⁺ magnetic cell sorting using anti-CD14 magnetic beads (Miltenyi Biotech, Bergisch Gladbach, Germany) according to the manufacturer’s instructions. Following CD14 magnetic enrichment, HC purity was assessed by flow cytometry and was consistently approximately 97%. HC identity was supported by expression of macrophage-lineage and HC-associated markers, including CD14, CD68, CD163, CD206, and CD209, as previously described (8, 13, 21). Although dedicated quantification of T-cell, NK-cell, trophoblast, endothelial, or stromal contamination was not performed for every sample in the present study, substantial lymphoid contamination is unlikely because placental villous tissue contains relatively few non-macrophage immune cells across gestation (51). If present, the most likely non-HC contaminating cells would be placental stromal/fibroblast-like cells; however, the CD14 enrichment strategy, culture conditions, and short-term 24-hour exposure design were optimized to maintain macrophage-lineage cells and limit fibroblast outgrowth.

Purified cells were cultured in complete RPMI medium containing 1× RPMI (Corning Cellgro, Corning, NY, USA), 10% fetal bovine serum (FBS; Optima, Atlanta Biologics), 2 mM L-glutamine (Corning), 1 mM sodium pyruvate (Corning), 1× non-essential amino acids (Corning), and antibiotics (penicillin, streptomycin, amphotericin B; Corning). Cells were maintained at 37 °C in a humidified incubator with 5% CO_2_.

### 5.3 Viral exposure

For HIV exposure, HCs were treated *in vitro* for 24 hours as previously described (13, 21, 24). Briefly, 5.0 × 10⁵ cells per well were seeded in 24-well plates (Corning) and infected at a multiplicity of infection (MOI) of 0.1 with the HIV-1 BaL strain for 24 hours at 37 °C. HIV-1 BaL was obtained through the NIH AIDS Reagent Program, Division of AIDS, NIAID, NIH (22). HIV-1 BaL is a well-characterized CCR5-tropic HIV strain originally isolated from infant lung tissue (52), and commonly used to model HIV infection and immune activation in monocyte/macrophage-lineage cells. Given that HCs are placental macrophages, BaL provides a biologically relevant model for assessing macrophage-tropic HIV exposure at the maternal–fetal interface.

For CMV exposure, HCs were exposed at an MOI of 0.1 for 24 hours at 37 °C to the human CMV TB40/E-GFP strain. This virus was originally generated by Dr. Christian Sinzger’s laboratory at Ulm University and was subsequently provided to Dr. Don Diamond at City of Hope, who shared the stock with our laboratory. CMV stocks were generated by propagating the virus in ARPE-19 cells.

Viral exposure was performed at an MOI of 0.1 for 24 hours to capture acute HC transcriptional and cytokine responses rather than prolonged multi-day viral replication. MOI refers to infectious viral units added per target cell; however, MOI values are not directly interchangeable between HIV-1 and HCMV because the viruses differ in particle-to-infectivity ratios, replication kinetics, cellular tropism, and stock quantification methods. Therefore, the selected MOI was used to standardize exposure within each viral system rather than to imply equivalent biological infectivity across viruses.

Mock-exposed cultures consisted of HCs cultured in the same complete RPMI medium and under the same 24-hour conditions as virus-exposed cultures, but without addition of viral inoculum. Virus-free matched producer-cell supernatant was not added to the mock condition.

Viral exposure and infection under the same or comparable experimental conditions have been previously evaluated by our group (13, 21, 24). HIV-1 BaL exposure/infection was assessed using p24 ELISA and HIV env/gag RNA qPCR, whereas HCMV TB40/E-GFP infection was assessed by GFP reporter expression. Under the MOI used in these experiments, approximately 40% of HCs were GFP-positive in prior validation experiments.

### 5.4 RNA sequencing and preprocessing

Total RNA was extracted 24 hours post-exposure using RNeasy (Qiagen), according to the manufacturer’s instructions. RNA quality was assessed using a NanoDrop and a Bioanalyzer; only samples with an RNA integrity number (RIN) > 7 were included. Libraries (150-bp paired-end) were prepared and sequenced by BGI, targeting ≥30 million reads per sample. Reads were quality-filtered using SOAPnuke (53) and aligned to the human reference genome GRCh38 using HISAT2 (54). Gene-level expected counts and TPMs were obtained with RSEM (55). Low-count genes were removed, retaining genes with counts per million (CPM) > 1 in at least two samples. Counts were normalized with TMM (edgeR), then transformed with voom (limma) with precision weights (56–58). For visualization (e.g., principal component analysis, PCA), we used voom-transformed values from the top 5,000 most variable genes.

### 5.5 Differential expression analysis

Differential expression was assessed using limma-voom. A group-based linear model was fit to the normalized expression matrix using the combined gestational-stage and viral-exposure condition as the design factor. The model included six experimental groups: early/mid mock-exposed HCs, early/mid HIV-exposed HCs, early/mid CMV-exposed HCs, term mock-exposed HCs, term HIV-exposed HCs, and term CMV-exposed HCs. Linear contrasts were used to test virus effects within gestational stage, gestational-stage effects within each exposure condition, and direct virus-versus-virus differences within gestational stage. The contrasts included early/mid CMV versus early/mid mock-exposed, early/mid HIV versus early/mid mock-exposed, term CMV versus term mock-exposed, term HIV versus term mock-exposed, early/mid mock-exposed versus term mock-exposed, early/mid CMV versus term CMV, early/mid HIV versus term HIV, term CMV versus term HIV, and early/mid CMV versus early/mid HIV. For direct CMV-versus-HIV contrasts, positive log_2_FC values indicate higher expression in CMV-exposed HCs, whereas negative log_2_FC values indicate higher expression in HIV-exposed HCs. Moderated statistics were computed using empirical Bayes shrinkage. P-values were adjusted using the Benjamini–Hochberg method; genes were considered differentially expressed at FDR < 0.05 with an additional threshold of |log_2_FC| > 1.

### 5.6 Gene-set enrichment and pathway annotation

Hallmark gene sets from the Molecular Signatures Database (MSigDB) were tested using FGSEA on full-ranked t-statistics (59, 60). Gene sets with FDR < 0.05 were considered significantly enriched. Over-representation analysis (ORA) was performed using Reactome, with the set of genes tested in the limma model as the background universe (61). For the direct CMV-versus-HIV contrasts, FGSEA and Reactome analyses were interpreted directionally, with positive NES indicating enrichment in CMV-exposed HCs and negative NES indicating enrichment in HIV-exposed HCs.

### 5.7 Weighted gene co-expression analysis

Co-expression modules were inferred with CEMiTool using voom-transformed expression values (62). Default filtering parameters were applied. Module activity was summarized per condition using Normalized Enrichment Score (NES) based on a multi-group reference. Module annotation was performed by Reactome ORA (FDR < 0.05). We report modules M1–M11, their leading biological themes, and their directionality across conditions.

### 5.8 Placenta-enriched cross-reference

To assess whether virus-responsive genes in HCs intersect placenta-specific biology, we used the Human Protein Atlas (HPA) tissue expression resource (43, 44). HPA tables for the placenta were downloaded, and genes were mapped to our DEGs. For each gestational stage (early/mid-gestation, term-gestation) and each contrast (HIV vs mock-exposed, CMV vs mock-exposed), we intersected the DEG lists (limma-voom; FDR < 0.05 and |log_2_FC| > 1) with the HPA placenta categories.

### 5.9 Cytokine profiling

Supernatants were collected from cultures seeded with equal numbers of purified HCs per condition (5 × 10⁵ cells/well) after 24-hour exposure. Supernatants were clarified by centrifugation prior to Luminex analysis. The six conditions assayed were early/mid-gestation HIV, term-gestation HIV, early/mid-gestation CMV, term-gestation CMV, early/mid-gestation mock-exposed, and term-gestation mock-exposed HCs. Cytokines were measured using a Luminex™ 200 system (Eve Technologies, Human Focused 15-Plex Discovery Assay^®^). The panel included GM-CSF, IFN-γ, IL-1β, IL-1Ra, IL-2, IL-4, IL-5, IL-6, IL-8, IL-10, IL-12p40, IL-12p70, IL-13, MCP-1, and TNF-α; assay sensitivities ranged from 0.14 to 5.39 pg/mL. Cytokine concentrations were reported as pg/mL and were not further normalized to viable cell number after the 24-hour exposure.

For each cytokine, we performed a Kruskal–Wallis test across the six conditions, followed by Dunn’s *post-hoc* pairwise comparisons with Bonferroni correction. Adjusted p-value < 0.05 was considered statistically significant, whereas 0.05 ≤ adjusted p-value < 0.07 was treated as a trend. Box plots display group distributions, with significance annotated as * 0.05 ≤ adj-p < 0.07, ** adj-p < 0.05, and *** adj-p < 0.01. All statistical analyses were conducted in R.

## Data Availability

All data generated or analyzed during this study are publicly available in the Gene Expression Omnibus (GEO) database under the accession number GSE274414.

## Supplementary material

Supplementary Figure 1 Differentially expressed genes and FGSEA results for comparison CMV term vs HIV term. (A) Volcano plot showing differential gene expression for the direct term CMV versus term HIV comparison. Positive log_2_FC values indicate genes higher in term CMV-exposed HCs, whereas negative log_2_FC values indicate genes higher in term HIV-exposed HCs. Differentially expressed genes were defined using FDR < 0.05 and |log_2_FC| > 1. (B) Hallmark FGSEA for the direct term CMV versus term HIV comparison. Positive NES indicates enrichment in term CMV-exposed HCs, whereas negative NES indicates enrichment in term HIV-exposed HCs.

Supplementary Table 1 Sample and donor metadata for Hofbauer cell RNA-seq. This table lists all samples included in the RNA-seq analyses, with sample IDs, virus exposure (mock-exposed, HIV, CMV), gestational stage (early/mid vs term), combined experimental class and batch assignment.

Supplementary Table 2 Differentially expressed genes across all contrasts. Long-format table with all genes meeting FDR < 0.05 and |log_2_FC| > 1 in any RNA-seq contrast. Contrasts include virus vs mock-exposed within each gestational stage, early/mid vs term within each exposure condition, and direct CMV versus HIV comparisons within term and early/mid gestation.

Supplementary Table 3 Hallmark gene set enrichment results (FGSEA) for all contrasts. Normalized enrichment scores (NES), p-values, and FDR values for all MSigDB Hallmark gene sets tested using FGSEA on ranked limma t-statistics. The table includes virus versus mock-exposed comparisons, gestational-stage comparisons within exposure condition, and direct CMV versus HIV comparisons.

Supplementary Table 4 Reactome over-representation analysis of shared up-regulated genes in term CMV and term HIV. Reactome pathway enrichment for the 85 genes up-regulated in both term CMV vs term mock-exposed and term HIV vs term mock-exposed.

Supplementary Table 5 Metadata for Hofbauer cell cultures analyzed by Luminex. Sample-level information for the cytokine profiling experiment.

Supplementary Table 6 Cytokine concentrations and statistical comparisons from Luminex assays. Concentrations (pg/mL) of GM-CSF, IFN-γ, IL-1β, IL-1Ra, IL-2, IL-4, IL-5, IL-6, IL-8, IL-10, IL-12p40, IL-12p70, IL-13, MCP-1, and TNF-α measured in supernatants from early/mid-gestation and term-gestation Hofbauer cell cultures under HIV, and CMV and mock exposition conditions.

Supplementary Table 7 Reactome analysis for comparison CMV term vs HIV term. Reactome pathway analysis for the direct term CMV versus term HIV comparison. Reactome FGSEA and/or over-representation results for genes and ranked statistics from the direct term CMV versus term HIV comparison. Positive NES indicates enrichment in term CMV-exposed HCs, whereas negative NES indicates enrichment in term HIV-exposed HCs.
