# Supplementary figures and images for: Distinct immune responses to HIV and CMV in Hofbauer cells across gestation highlight evolving placental immune dynamics
Source: Front Immunol. 2026 Jun 9;17:1832988. doi: 10.3389/fimmu.2026.1832988 (PMC13286909; doi:10.3389/fimmu.2026.1832988)

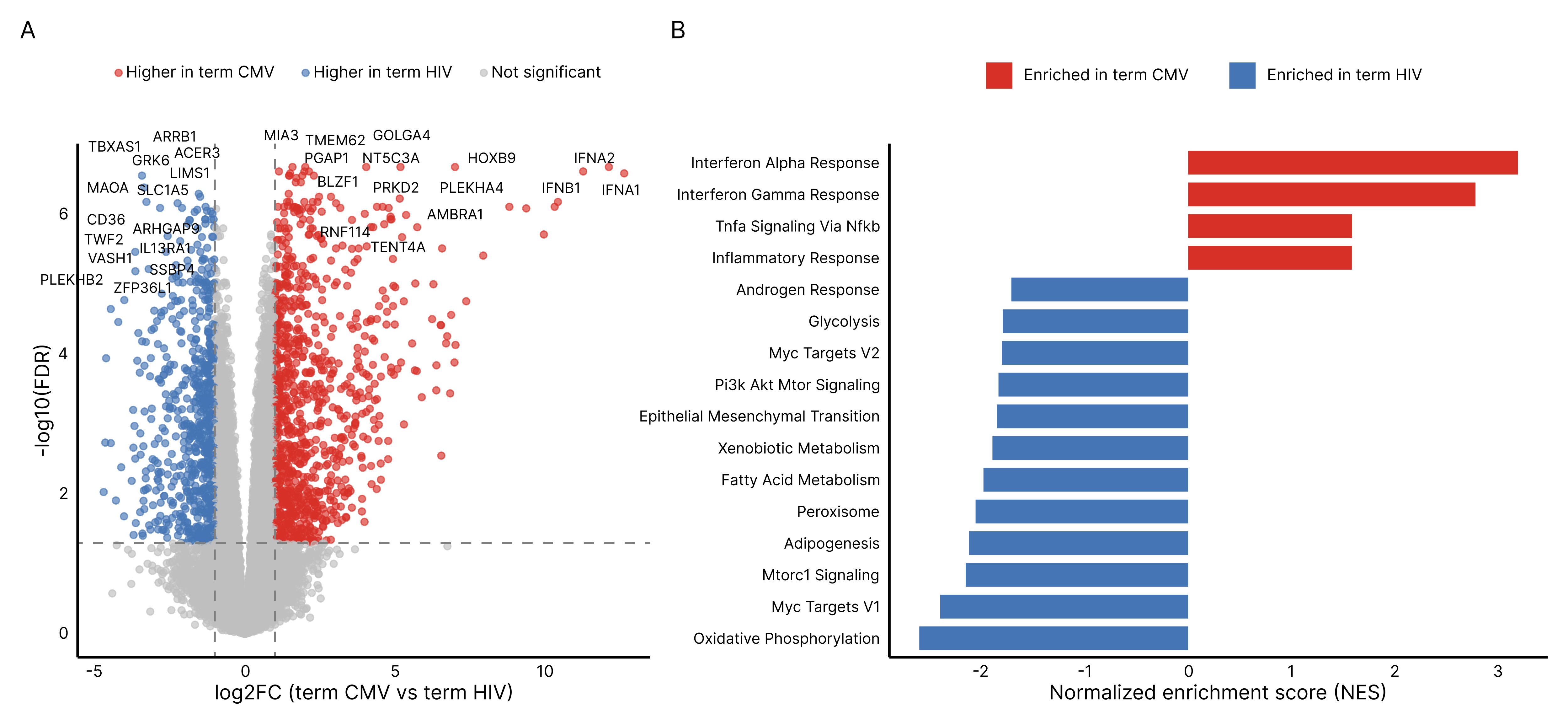

Supplement: Supplementary Figure 1 — Differentially expressed genes and FGSEA results for comparison CMV term vs HIV term. (A) Volcano plot showing differential gene expression for the direct term CMV versus term HIV comparison. Positive log2FC values indicate genes higher in term CMV-exposed HCs, whereas negative log2FC values indicate genes higher in term HIV-exposed HCs. Differentially expressed genes were defined using FDR < 0.05 and |log2FC| > 1. (B) Hallmark FGSEA for the direct term CMV versus term HIV comparison. Positive NES indicates enrichment in term CMV-exposed HCs, whereas negative NES indicates enrichment in term HIV-exposed HCs. [file Image1.jpeg]
